# Case Report and Review of the Pathophysiology and Therapeutics of Adult Hematohidrosis

**DOI:** 10.7759/cureus.36187

**Published:** 2023-03-15

**Authors:** Juvarez U Ogbuneke, John C Allen

**Affiliations:** 1 Internal Medicine, Baptist Memorial Hospital, Oxford, USA; 2 Hematology and Oncology, Baptist Memorial Hospital, Oxford, USA

**Keywords:** eccrine sweat glands, rare condition, bloody sweat, hematology disorders, hematohidrosis

## Abstract

Hematohidrosis is an extremely rare condition characterized by the oozing or secretion of blood through intact skin and mucosa, particularly through eccrine glands. Although there is not much literature available on the condition, examples of Hematohidrosis include the crying and sweating of blood. The fluid may have a bloody tinge or may be frank blood. The anomaly has no identifiable etiology, and patients generally present in a good state of health. In this report, we present a 19-year-old female who had weekly occurrences of bloody diaphoresis that had been present consistently for one year. During her presentation at the hematology clinic, she was investigated thoroughly for alternative causes, but none were found. The patient was diagnosed with hematohidrosis and was offered treatment with propranolol, which she declined. She continues to follow up routinely in the hematology clinic with persistent symptoms.

## Introduction

Hematohidrosis is a very rare condition that has presented itself in many places throughout history. One notable area would be in religious texts while others are the rarely documented medical occurrences or even in the descriptions of Leonardo da Vinci [1]. The etiology of hematohidrosis is unclear, and it is believed to manifest in extreme stress [2]. The hypothesized mechanism of action is that the bleeding is due to the rupture of the tiny blood vessels of the skin's dermal capillaries [2]. Although there are few cases, the highest distribution of hematohidrosis lies in Asia [1].

## Case presentation

**Figure 1:**
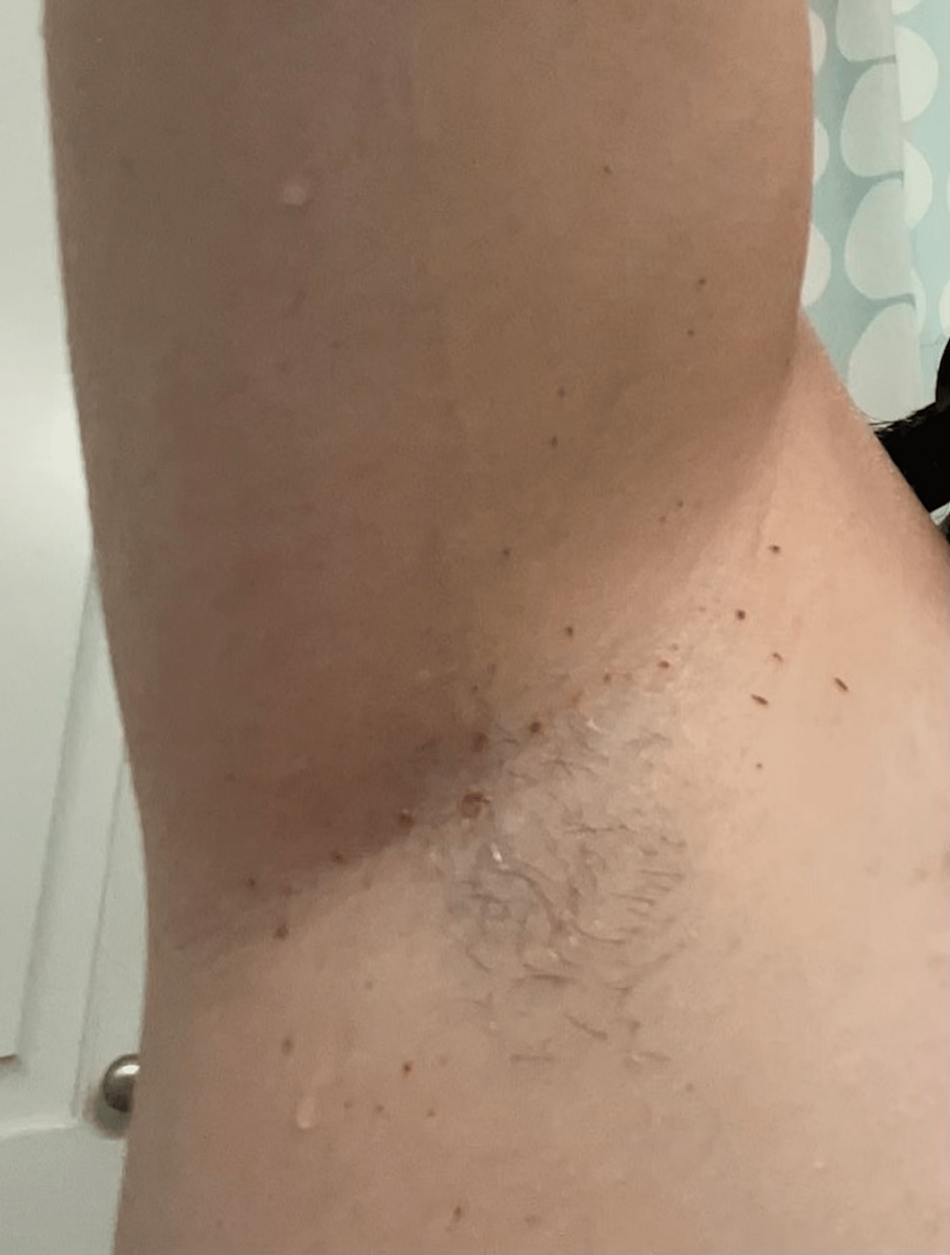
Hematohidrosis of the axillae

A 19-year-old white female with a significant past medical history of epilepsy and juvenile absence seizures presented to the hematology clinic with the complaint of “sweating blood.” The patient had experienced the symptoms intermittently as a younger child when she would sweat blood from her forehead. Since that time, she had not experienced any further episodes until one year prior to her presentation to our clinic. Notably, the patient had never undergone diagnostic testing for her condition prior to arrival at the clinic. The patient reported that over the previous year, she had experienced consistently bloody sweat from intact skin, most prominently in the bilateral axillary regions, which progressively spread distally towards the forearm (Figure 1). She experienced an exacerbation of the symptoms only in times of exertion, most prominently when exercising. On physical examination, the patient was a well-nourished female without any excoriations, ecchymoses, petechiae, or lymphadenopathy. She reported no family history of any bleeding disorders or similar manifestations. She was taking no potential causative medications. An extensive blood panel workup was taken (complete blood count, prothrombin time, partial thromboplastin time, Von Willebrand’s, platelet aggregation w/ Ristocetin panel, complete metabolic panel), which resulted in a negative result, effectively ruling out a plethora of other bleeding disorders. In follow-up, the patient continued to have symptoms of bloody sweat present on the forearms as well as in the axillary regions. Due to the extensive and unrevealing workup accompanied by her symptoms, the patient was clinically diagnosed with hematohidrosis without an underlying bleeding disorder. She was offered education and treatment with propranolol but declined treatment. She continues to follow up at the clinic and continues to experience the same symptoms.

## Discussion

Hematohidrosis is an extremely rare condition that is characterized by the spontaneous excretion of blood through intact skin and is not caused by any type of trauma [1]. Although there is no established etiology, there have been many proposed hypotheses of the pathophysiology of the disease. Many individuals believe that the capillary network surrounding the eccrine sweat glands ruptures due to stress, thus excreting blood into the sweat glands [2]. As great stress occurs, the net-like blood vessels constrict, and when the stressor passes, the vessels dilate to the point of rupture. As sweat accumulates in the gland, it pushes the blood droplets to the surface, forming a mix of sweat and blood [2]. Although there is not much data circulating in regard to the treatment of hematohidrosis, current therapeutic options found to be successful have been the use of beta-blockers. It has been reported that 83% of hematohidrosis patients present at age 18 and younger [3]. The patient in our case was a 19-year-old female, which is older than the usual age presentation of the disease [3], though notably, she reported symptoms dating back to a much younger age. Uniquely, this case involved an American Caucasian female, though hematohidrosis has been rarely reported in this part of the world. Out of 36 multinational cases of hematohidrosis, the highest number of cases were in Asia, specifically, India [1]. Psychological stress has been associated with a strong connection to hematohidrosis [4-6], but in this patient, there was no evidence of psychological or psychiatric stress.

## Conclusions

Hematohidrosis is a distressing experience for many patients and their loved ones. Due to the visualization of bleeding, there can be surreptitious diagnostics, which can affect the morbidity or mortality of patients. Hematohidrosis is a rare condition without a lot of information. The more cases reported the more information will be provided to the global medical community about this benign condition.
